# Epigenetic modulation of the drug resistance genes MGMT, ABCB1 and ABCG2 in glioblastoma multiforme

**DOI:** 10.1186/1471-2407-13-617

**Published:** 2013-12-31

**Authors:** Moritz C Oberstadt, Sandra Bien-Möller, Kerstin Weitmann, Susann Herzog, Katharina Hentschel, Christian Rimmbach, Silke Vogelgesang, Ellen Balz, Matthias Fink, Heike Michael, Jan-Philip Zeden, Henrike Bruckmüller, Anneke N Werk, Ingolf Cascorbi, Wolfgang Hoffmann, Dieter Rosskopf, Henry WS Schroeder, Heyo K Kroemer

**Affiliations:** 1 Department of Pharmacology, Ernst-Moritz-Arndt-University, Greifswald, Germany; 2 Department of Epidemiology of Health Care and Community Health, Ernst-Moritz-Arndt-University, Greifswald, Germany; 3 Department of Neuropathology, Ernst-Moritz-Arndt-University, Greifswald, Germany; 4 Department of Neurosurgery, Ernst-Moritz-Arndt-University, Greifswald, Germany; 5 Department of Experimental and Clinical Pharmacology, University Hospital Schleswig-Holstein, Kiel, Germany

**Keywords:** Glioblastoma multiforme, MGMT, Drug resistance, DNA methylation

## Abstract

**Background:**

Resistance of the highly aggressive glioblastoma multiforme (GBM) to drug therapy is a major clinical problem resulting in a poor patient’s prognosis. Beside promoter methylation of the *O*^*6*^*-methylguanine-DNA-methyltransferase (MGMT)* gene the efflux transporters ABCB1 and ABCG2 have been suggested as pivotal factors contributing to drug resistance, but the methylation of *ABCB1* and *ABCG2* has not been assessed before in GBM.

**Methods:**

Therefore, we evaluated the proportion and prognostic significance of promoter methylation of *MGMT*, *ABCB1* and *ABCG2* in 64 GBM patient samples using pyrosequencing technology. Further, the single nucleotide polymorphisms MGMT C-56 T (rs16906252), ABCB1 C3435T (rs1045642) and ABCG2 C421A (rs2231142) were determined using the restriction fragment length polymorphism method (RFLP). To study a correlation between promoter methylation and gene expression, we analyzed MGMT, ABCB1 and ABCG2 expression in 20 glioblastoma and 7 non-neoplastic brain samples.

**Results:**

Despite a significantly increased *MGMT* and *ABCB1* promoter methylation in GBM tissue, multivariate regression analysis revealed no significant association between overall survival of glioblastoma patients and *MGMT* or *ABCB1* promoter methylation. However, a significant negative correlation between promoter methylation and expression could be identified for MGMT but not for ABCB1 and ABCG2. Furthermore, *MGMT* promoter methylation was significantly associated with the genotypes of the MGMT C-56 T polymorphism showing a higher methylation level in the T allele bearing GBM.

**Conclusions:**

In summary, the data of this study confirm the previous published relation of *MGMT* promoter methylation and gene expression, but argue for no pivotal role of *MGMT*, *ABCB1* and *ABCG2* promoter methylation in GBM patients’ survival.

## Background

Glioblastoma multiforme (GBM) is still the most frequent primary brain tumor in adults and is characterized by a highly aggressive phenotype [1]. Despite advances in therapy, glioblastoma remains associated with poor prognosis and an overall survival time of about 1 year [2]. A major underlying factor is resistance to different chemotherapeutics. Several chromosomal, genetic and epigenetic alterations were identified in GBM [3], but the clinical value of the most glioma-associated molecular aberrations remained unclear [4]. However, a significant prognostic impact could be shown for the O^6^-methylguanine-DNA-methyltransferase (MGMT). The MGMT functions as a DNA repair enzyme, which repairs alkylating lesions of the DNA by removing mutagenic adducts from the O6 position of guanine, e.g. caused by the chemotherapeutic agent temozolomide [5]. Hence, it confers drug resistance and the therapeutic response to alkylating agents is improved in tumor cells expressing low levels of MGMT [5]. Furthermore, *MGMT* promoter methylation was demonstrated to result in decreased MGMT expression and correlates with a survival benefit in glioblastoma patients treated with alkylating chemotherapeutics such as temozolomide [6].

Expression and activity of the efflux transporters ABCB1 and ABCG2 have also been suggested as pivotal factors contributing to drug resistance by increasing the efflux of chemotherapeutic compounds in the setting termed “multidrug resistance”. These ATP-binding cassette transporters (ABC transporters) belong to a superfamily of membrane pumps that use ATP hydrolysis to efflux various endogenous compounds and drugs outside the cell. ABCB1 was shown to be expressed both in low-grade glioma and high-grade glioma such as glioblastoma [7] and ABCG2 was found to be expressed in glioma stem cells as well as in endothelial cells of the large vessels of glioma tissue [5]. For both ABCB1 and ABCG2 an inverse correlation between the methylation status of Cytidine phosphate Guanosine (CpG) sites at the promoter region and the transporter expression was demonstrated [8,9]. Furthermore, *ABCB1* promoter methylation is associated with the *ABCB1 C3435T* polymorphism which again influences the ABCB1 expression [10]. Similarly, for ABCG2 an association of the *ABCG2 C421A* polymorphism with both the transport function and expression of the efflux transporter was shown [11,12].

*ABCB1* and *ABCG2* promoter methylation have not been assessed in glioblastoma patients before. We therefore investigated promoter methylation of *ABCB1* and *ABCG2* in 64 glioblastoma patients using the pyrosequencing technology, which allows unequivocal quantification of the methylation status, and used *MGMT* promoter methylation as positive control.

In our study we found a significantly increased *MGMT* and *ABCB1* promoter methylation in GBM tissue but couldn’t demonstrate any association of *MGMT*, *ABCB1* or *ABCG2* promoter methylation with overall survival of glioblastoma patients in multivariate Cox models adjusted for potential risk factors (gender and age) and stratified on the variable therapy (temozolomide vs. no temozolomide). However, we found a significant negative correlation between *MGMT* promoter methylation and MGMT expression and a significant association between *MGMT* methylation and the *MGMT C-56 T* polymorphism.

## Methods

### Patient samples

Malignant glioblastoma samples (n = 64) were obtained from patients who had undergone tumor resection at the Clinic of Neurosurgery of the University of Greifswald, Germany. Tumor samples were collected between 2003 and 2009 from patients with newly diagnosed glioblastoma who had received no antitumoral therapy before sample collection. Additionally, relapses of 17 of these patients were collected. For investigation of methylation status, fresh frozen human glioblastoma tissue samples (n = 4) and paraffin-embedded glioblastoma sections (n = 60) were analyzed by pyrosequencing, which is described as a highly reproducible method for quantification of *MGMT* methylation in both formalin-fixed paraffin-embedded and fresh frozen samples [13,14]. Samples from 11 of the 64 GBM patients have been available for mRNA expression analysis and 9 further GBMs have been added to investigate the mRNA expression in a total of 20 GBM patients.

All tumor samples were histologically classified by a neuropathologist at the Department of Pathology of the University of Greifswald according to the WHO criteria of tumors of the nervous system using formalin-fixed, paraffin-embedded specimens. Clinico-pathological features of the analyzed patients are summarized in Table 1. All investigations described in this study were approved by the Ethics Committee of the University of Greifswald, Germany.

**Table 1:** Clinico-pathological features of the analyzed patients

**Characteristic**	**Age [Years]**	
Median age at diagnosis	61.6	
Range [Min.-Max.]	40.2 - 79.9	
Patients with temozolomide therapy		
Median age at diagnosis	59.2	
Patients without temozolomide therapy		
Median age at diagnosis	64.0	
Characteristic	Number of patients	% of patients
Age classes		
<50 years	11	17.2
50 - 60 years	18	28.1
60 - 70 years	20	31.3
>70 years	15	23.4
Sex		
Male	39	60.9
Female	25	39.1
Pathohistology		
Glioblastoma multiforme	64	
Relapses of primary glioblastoma multiforme	17	
Therapy		
Only Radiotherapy	11	17.2
Radiotherapy and temozolomide	45	70.3
No adjuvant therapy	6	9.4
No therapy data applicable	2	3.1
Overall survival (OS)		
Median [Days]	459	
Range [Min.-Max.]	34 - 1954	
1-year survival	38	59.4
2-year survival	9	14.1
OS of patients with temozolomide therapy		
Median [Days]	515	
Range [Min.-Max.]	95 - 1954	
OS of patients without temozolomide therapy		
Median [Days]	87	
Range [Min.-Max.]	34 - 701	
Vital status at study end (30.06.2009)		
Dead	47	73.4
Alive	17	26.6

### DNA Isolation

Genomic DNA (gDNA) was isolated from fresh frozen tumor samples or formalin-fixed, paraffin-embedded glioblastoma sections using the NucleoSpin® Tissue Kit (Macherey-Nagel, Düren, Germany) according to the manufacturer’s instructions. 2–5 slices à 5 μm of the formalin-fixed, paraffin-embedded glioma tissue sections were used per sample. Concentrations of the isolated genomic DNA were determined using a NanoDrop 1000 Spectrophotometer (PEQLAB, Erlangen, Germany).

### Bisulfite Treatment and PCR Amplification

For evaluation of the promoter methylation status of *MGMT*, *ABCB1* and *ABCG2* 1800 ng of the isolated gDNA per sample were bisulfite treated using the EpiTect® Bisulfite Kit (Qiagen, Hilden, Germany) following the manufacturer’s instructions. The bisulfite treated DNA was subjected to PCR amplification of the specific promoter regions of *MGMT*, *ABCB1* and *ABCG2* gene by the use of primer sets designed to amplify sequences containing CpG sites to be investigated (Table 2). The detailed conditions for the PCR amplification of the promoter region of interest are summarized in the Additional file 1 with the Figures S1-S3.

**Table 2:** Primer sequences used for methylation analysis

**Gene symbol**	**GenBank accession**	**Forward primer 5′- > 3′**	**Reverse primer 5′- > 3′**	**Sequencing primer 5′- > 3′**	**Amplicon size (bp)**
MGMT	X61657.1	YGYGTTTYGGATATGTTGGGATAG	Biotin -AACRAAA CRACCCAAACACTCA	GGATAGTTYGYGTTTTTAGA	115
ABCB1	AH002875.1	GTGGGTGGGAGGAAGTAT	Biotin -AAATCTC CAACATCTCCAC	GGGTAAAGTTTAGAA	125
ABCG2	AH011213.2	TGATTGGGTAATTTGTGTGTTAGTG	Biotin -AAATAAA CCAAAATAATTA ACTAC	TTGTGATTGGGTAATTTGTG	147

### Pyrosequencing for promoter methylation analysis

Pyrosequencing analysis was performed on the PSQ™ 96MA System (Biotage, Uppsala, Sweden). Methylation of target CpGs was assessed by determining the ratio of cytosine to thymine incorporated during pyrosequencing. Cytosine incorporation indicated a methylated CpG and thymine incorporation an unmethylated CpG. Quantification of the methylation status was performed using the provided software from PSQ™ 96MA System (Biotage, Uppsala, Sweden).

Five CpG methylation sites were investigated for *MGMT* promoter methylation, two for *ABCB1* promoter methylation and three for *ABCG2* promoter methylation. The average percentage methylation of the different CpG sites of each gene promoter was calculated and used in all analyses. During the establishing process of the methylation assays, the analytical sensitivity and quantitative accuracy of the three methylation assays have been assessed. We correlated the methylation results for the first CpG site of ABCB1 (Additional file 1: Table S1A), ABCG2 (Additional file 1: Table S1B) and MGMT (Additional file 1: Table S1C) methylation assays of three independent measurements. These same 19 samples measured in triplicates determined a high quantitative accuracy of the assays with high significant (*** p < 0.001) Spearman correlation coefficients between 0.88 and 0.99 (Additional file 1: Tables S1A-C).

### Methylation-specific PCR (MSP)

1.8 μg DNA has been bisulfite-converted using the EpiTect® Bisulfite Kit (Qiagen, Hilden, Germany). 2 μl of the bisulfite-converted DNA was amplified in a PCR consisting of 20 pmol of primers (Eurofins MWG Operon, Ebersberg, Germany), 1.25 mM MgCl_2_, 10x Reaction buffer, 1.5 units Taq-Polymerase and 200 μM dNTPs (all Invitrogen, Karlsruhe, Germany). The thermal cycling conditions used were as follows: 95°C for 10 min, and 40 cycles of 95°C for 45 sec, 52°C for 50 sec, 72°C for 1 min with a final extension of 72°C for 10 min. Two μl of the amplified first-round product was used for second round of amplification with 20 pmol of primers (Eurofins MWG Operon, Ebersberg, Germany), 1.25 mM MgCl_2_, 10x Reaction buffer, 1.5 units Taq-Polymerase and 200 μM dNTPs (all Invitrogen, Karlsruhe, Germany). The following thermal cycling conditions were followed: 95°C for 10 min, and 20 cycles of 95°C for 45 sec, 65°C for 25 sec, 72°C for 30 sec with a final extension of 72°C for 10 min. The amplified products were run on a 2% agarose gel with an expected size of 81 bp for methylated product and 93 bp for an unmethylated product.

We analyzed the agarose gel bands using the KODAK Gel Logic 200 Imaging System (Eastman Kodak Company, Rochester, NY, USA) (Additional file 1: Figure S8). Our corresponding pyrosequencing results for MGMT are included in Additional file 1: Table S2. To validate the performance of the MSP conditions chosen, methylated and unmethylated standard samples provided from the *EpiTect PCR Control Set* (Qiagen, Hilden, Germany) have been used as controls which showed the expected bands only in either the methylated or unmethylated PCR (Additional file 1: Figure S8). However, beside U87MG glioblastoma cells as a methylated reference [15] and LN18 glioblastoma cells, we chose a spectrum of differently methylated GBM samples of the pyrosequencing analysis: two strong, two middle and two unmethylated GBM samples for assay comparison. Even though it is difficult to directly compare the qualitative method of MSP with the quantitative method of pyrosequencing, it is still visible, that those three glioblastoma samples (GBM1, GBM3, and GBM6) with the most intensive methylated bands in MSP show in addition to U87MG cells the three highest methylation percentages in the pyrosequencing analysis (28.2%, 61.21%, and 74.74%), indicating more or less comparable results of both methylation detection methods.

### Quantitative Real-Time PCR

Total RNA was isolated from 20 human fresh frozen glioblastoma samples and 7 normal brain tissue samples (frontal/temporal lobes) using the PeqGold RNAPure™ reagent protocol (Peqlab Biotechnologie, Erlangen, Germany), which allows (based on the guanidinisothiocyanat) the dissociation of cells and inactivation of RNases and other enzymes at the same time. The provider of RNAPure guarantees optimal purity and high rates of yields of non-degraded RNA. Subsequently, RNA was measured photometrically at the wavelength of 260 nm using the Nano Drop™ 1000 Spectrophotometer from PEQLAB (Erlangen) to get information about the purity. 1 μl of each sample was applied. Beside the concentration of the RNA, indicated in μg/μl, the purity ratios 260/280 and 260/230 were determined. It was proven, that the purity ratio (260/280) of our samples accounts for 1.8 to 2.0 (2.2 for the ratio 260/230). RNA was further always placed on ice to avoid degradation and long-time storing of the RNA was performed at -80°C.

500 ng of total RNA were used for cDNA synthesis with the High Capacity cDNA Reverse Transcription Kit (Applied Biosystems, Foster City, CA) in a 20 μl reaction volume. Real-time PCR was performed with 10 ng final concentration of cDNA using the ABI Prism 7900 Sequence Detection System (Applied Biosystems, Foster City, CA). cDNA was amplified using Assays on Demand for MGMT (Hs01037698_m1), ABCB1 (Hs00184491_m1), and ABCG2 (Hs01053790_m1), all conjugated with fluorochrome 5-carboxyfluorescein (FAM), and 18S rRNA (Predeveloped TaqMan Assay Reagent, catalog no.: 4319413E, Applied Biosystems, Foster City, CA) conjugated with fluorochrome VIC (Applied Biosystems). Applied Biosystems guarantee maximum and equivalent amplification efficiency as well as specificity of all TaqMan® Assays-on-Demand Gene Expression Products (Application Note, Applied Biosystems: Amplification Efficiency of TaqMan® Assays-On-Demand™ Gene Expression Products). Further, only assays with exon junction spanning probes were selected in order to avoid amplification of contaminating genomic DNA. The analysis of the amplification efficiencies of our used PCR assays by measuring a serial dilution of selected cDNA showed a PCR efficiency of about 90% for all assays (Additional file 1: Figure S4A-F) allowing us to analyze the expression of our target genes by the ∆∆C_T_-method. Thus, quantification was performed with the comparative ∆∆C_T_-method. For the analysis of the quantitative RT-PCRs using the delta Ct-method we set the expression value of each GBM sample against the mean expression value of all analyzed control brain samples. Thus, the target gene expression in the GBM samples represents a multiple of the target expression in the control brain.

In addition to 18S rRNA we further analyzed the gene expression of TBP and GAPDH to validate their suitability as housekeeping genes in our samples. Using commercially available GAPDH and TBP assays (Applied Biosystems), we determined a similar distribution of values in 10 non-malignant brains, 97 GBM samples and 21 astrocytomas validating the expression measurements of MGMT, ABCB1 and ABCG2 based on normalization to the 18S rRNA content of our samples, as seen in the Additional file 1: Figure S7*.*

### Analysis of genetic variants

All patients were screened for *MGMT C-56 T* (rs16906252), *ABCB1 C3435T* (rs1045642) and *ABCG2 C421A* (rs2231142) gene polymorphisms using the polymerase chain reaction-restriction fragment length polymorphism (PCR-RFLP) method using the primers listed in Table 3. The detailed conditions for PCR-RFLP are described in the Additional file 1.

**Table 3:** Primer sequences used for genotyping

**Gene symbol**	**GenBank accession**	**Forward primer 5′- > 3′**	**Reverse primer 5′- > 3′**	**Amplicon size (bp)**
MGMT	X61657.1	CTAGAACGCTTTGCGTCCCGAC	CAACACCTGGGAGGCACTTG	231
ABCB1	AH002875.1	TGTTTTCAGCTGCTTGATGG	AAGGCATGTATGTTGGCCTC	197
ABCG2	AH011213.2	TGTTGTGATGGGCACTCTGATG	ATCAGAGTCATTTTATCCACAC	222

### mRNA expression of the markers CD133, GFAP and PECAM in glioblastoma samples

To assess the content of tumor cells and endothelial cells we decided to measure GFAP as a marker of astrocytic cells, CD133 as marker for glioblastoma stem-like cells and PECAM (CD31) as endothelial marker in the glioblastoma and non-malignant brain tissue. The CD133, GFAP and PECAM expression in non-malignant brain, glioblastomas (GBM) and the glioblastoma cell line LN18 is shown in Additional file 1: Figure S6.1. The expression of CD133 is significantly elevated in GBMs compared to non-malignant brain samples, showing that glioma stem-like cells are probably more common in the tumors than in healthy brain. These findings support that most of the cells analysed in our GBM samples represent tumor cells [16]. Besides, GFAP and PECAM expression greatly vary between the glioblastoma samples, but are not significantly different to the non-malignant brain, indicating a similar number of astrocytes and especially endothelial cells in the tumor tissue. Thus, our findings of an altered methylation status in GBM compared to non-malignant brain are mostly based on tumor cells instead of endothelial cells.

Furthermore, we correlated the expression data of GFAP, CD133 and PECAM with MGMT, ABCB1 or ABCG2 expression. MGMT, ABCB1 and ABCG2 did not significantly correlate with either GFAP, CD133 or PECAM gene expression (Additional file 1: Figures S6.2, S6.3 and S6.4) except the slight, but significant correlation of ABCG2 and PECAM (Spearman’s r = 0.494, p = 0.037, Additional file 1: Figure S6.4C), which may be due to the known localization of ABCG2 in endothelial cells of the blood–brain and the blood-tumor barrier.

Nevertheless, an exact comparison to or quantification of the tumor cell content in relation to other cell types in the glioblastoma tissue does not seem possible since each individual tumor cell can hold a different pattern of gene expression and thus our expression analysis gives an insight into the tumor in its entirety but not into the individual cells that form the whole tumor mass.

### Statistical analysis

Methylation data were analyzed using the statistical programs SAS V 9.1 (SAS Institute Inc., Cary, NC, USA) and STATA (Intercooled Stata/SE 10.1). Frequencies were calculated for categorical data. Metric data were described using median and interquartile range as well as minimum and maximum values. Spearman correlation, Mann Whitney *U* test (comparison of two groups), Kruskal Wallis test (comparison of > 2 groups) and Fisher’s exact test were used for bivariate comparisons. A p-value of <0.05 was considered to indicate statistical significance. The multivariate Cox proportional hazard regression analysis was used to examine the association between the patient’s overall survival and mean methylation of *ABCB1*, *ABCG2* and *MGMT*, respectively, adjusted for potential risk factors including gender and age at diagnosis.

The duration of a patient’s overall survival (OS) was defined as the time from the first tumor detection until death or the end of the study (30.6.2009). Patients who were alive at the end of the study were included as censored data into the model. The variable “therapy” (with temozolomide vs. without temozolomide) did not fulfil the assumptions of proportionality and was excluded from the *a priori* defined model. This variable was used as strata variable instead. All predictors were dummy coded. Hazard ratios and 95% confidence intervals were estimated. In a sensitivity analysis we included (1.) mean percentage of methylation over the respective methylation sites as continuous variable and (2.) every single methylation site separately as continuous variable into the model. Furthermore two different Cox models were analyzed for patients treated with or without temozolomide, respectively.

## Results

### Clinico-pathological features of the analyzed patients

The study population comprised 64 patients with glioblastoma multiforme WHO°IV (GBM). For the correlation of the methylation degrees between primary tumors and relapses, 17 relapses of primary glioblastoma multiforme WHO °IV tumors were analyzed in comparison to the respective primary tumor.

Clinico-pathological features of all analyzed patients are summarized in Table 1. The therapy regime was in accordance to the current recommendations for the respective tumor entity. 17.2% (11 GBM) of patients were treated with only radiotherapy, 70.3% (45 GBM) were treated with radiotherapy and temozolomide, 9.4% (6 GBM) got no adjuvant treatment and for 3.1% (2 GBM) data of therapy modalities are missing. The median OS for all patients was 459 days (Min. 34 days, Max. 1954 days). The median OS for patients treated with temozolomide as part of their therapy was 515 days (Min. 95 days, Max. 1954 days), while the median age for patients not treated with temozolomide was 87 days (Min. 34 days, Max. 701 days). This difference in median OS between patients treated with (515 days) versus without temozolomide (87 days) was statistically highly significant in a bivariate analysis (p < 0.01).

### Methylation status, expression level and overall survival of glioblastoma patients

**Figure 1:**
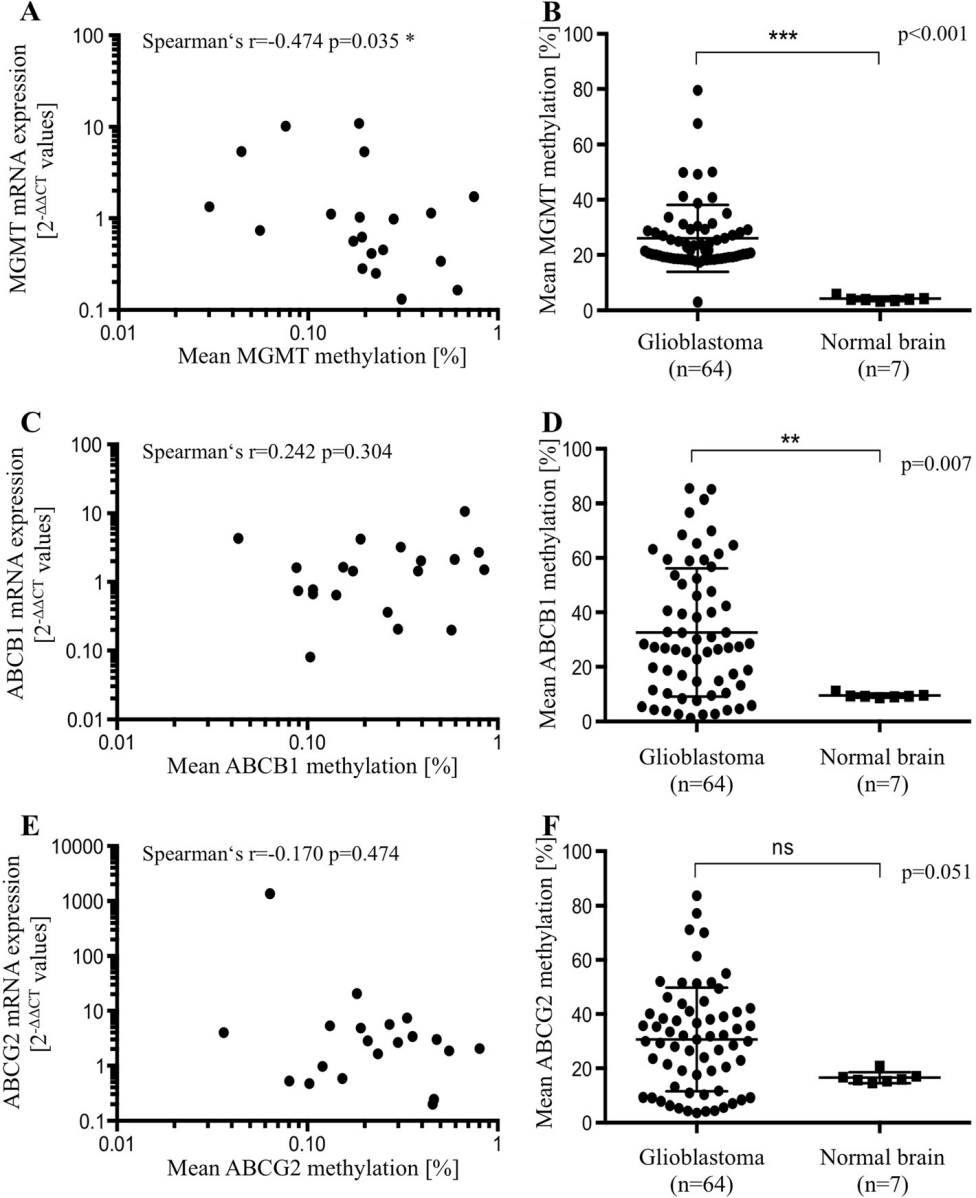
**Correlation analyses between MGMT, ABCB1 and ABCG2 mRNA expression and mean promoter methylation as well as comparison of MGMT, ABCB1 and ABCG2 promoter methylation between glioblastoma and non-malignant brain samples.** Correlation analysis of mRNA expression [2^-∆∆CT^] and promoter methylation [%] was performed for 20 GBM specimens **(A, C, E)**. Comparison of MGMT promoter methylation [%] between 64 GBM and 7 non-malignant brain specimens **(B, D, F) (A)** Correlation of MGMT mRNA expression and MGMT promoter methylation (Spearman’s rank correlation coefficient: -0.474; p = 0.035), **(C)** ABCB1 mRNA expression and ABCB1 promoter methylation (Spearman’s rank correlation coefficient: 0.242, p = 0.304), and **(E)** ABCG2 mRNA expression and ABCG2 promoter methylation (Spearman’s rank correlation coefficient: -0.170, p = 0.474). **(B)** Comparison of MGMT promoter methylation between GBM and non-malignant brain specimens (Mann–Whitney *U* test, p < 0.001), **(D)** ABCB1 promoter methylation between GBM and non-malignant brain samples (Mann–Whitney *U* test, p = 0.007), and **(F)** ABCG2 promoter methylation between GBM and non-malignant brain specimens (Mann–Whitney *U* test, p = 0.051).

Several studies predict *MGMT* promoter methylation as an important prognostic factor for clinical outcome of glioblastoma patients treated with temozolomide [6,17]. Therefore, we analyzed five CpG sites in the *MGMT* promoter, of which four CpG sites have been already investigated in a previous cutting-edge publication in the field [18]. Because *MGMT* methylation was suggested as a pivotal prognostic factor for OS of glioma patients who were treated with temozolomide [6,17], we established Cox models for all glioblastoma patients, patients treated with temozolomide as well as patients without temozolomide application, respectively. Continuous Cox models for the entire glioblastoma patient cohort (with and without temozolomide treated patients together), for the patients treated with temozolomide and for the patients treated without temozolomide did not show any significant overall survival difference dependent on the *MGMT* methylation level (Table 4). Also Dunn and colleagues used for their studies the method of pyrosequencing, but showed *MGMT* methylation as an independent prognostic factor associated with prolonged OS [19]. Thus, we analyzed the association of *MGMT* methylation and OS by dividing the *MGMT* methylation levels in the subgroups according to Dunn and colleagues by using our cut-off of 5.72% (mean normal brain ± 2 s.d.; first group: methylation level >5.72% - <20%; second group: methylation level >20% - <35%; third group: methylation level >35%, Additional file 1: Figure S5) [19]. However, this analysis displayed no significant difference in OS between the subgroups as well (Kruskal Wallis test, p = 0.9948). Because it is known, that *MGMT* methylation and expression are tightly linked [20] in the way that *MGMT* methylation leads to loss of MGMT expression [21], we analyzed this association in a subgroup of 20 GBM patients for which MGMT expression levels have been available. A significant negative correlation between *MGMT* methylation and expression could be identified (Spearman’s rank correlation coefficient: -0.474; p = 0.035; Figure 1A), indicating the downregulation of MGMT expression by methylation [21]. Furthermore, a highly significant elevated *MGMT* methylation has been detected for 64 GBM patient samples compared to 7 healthy brain samples (Mann Whitney test p < 0.001; Figure 1B).

**Table 4:** Multivariate analysis of MGMT promoter methylation and its association with the overall survival of GBM patients

**Variable**	**Haz. ratio**	**p-value**	**[95% Conf. Interval]**	
Sex				
male	1.488	0.238	0.769	2.876
(ref. female)	*1.259*	*0.602*	*0.530*	*2.992*
	**1.724**	**0.393**	**0.494**	**6.024**
Age				
50- <60 years	1.734	0.299	0.613	4.903
(ref. <50 years)	*1.648*	*0.394*	*0.523*	*5.192*
	**1.183**	**0.916**	**0.053**	**26.577**
Age				
60- <70 years	2.567	0.057	0.972	6.780
(ref. <50 years)	*3.242*	*0.039*	*1.061*	*9.901*
	**1.417**	**0.757**	**0.156**	**12.826**
Age				
≥70 years	6.427	0.001	2.194	18.824
(ref. <50 years)	*10.700*	*0.000*	*2.998*	*38.191*
	**2.442**	**0.445**	**0.247**	**24.152**
Mean methylation	0.988	0.315	0.964	1.012
level (continuous)	*0.975*	*0.121*	*0.945*	*1.007*
	**1.023**	**0.403**	**0.970**	**1.078**

Continuous multivariate Cox model regression analysis of MGMT promoter methylation and its association with the overall survival (OS) of the analyzed patients with glioblastoma multiforme, adjusted for potential risk factors including sex and age at diagnosis and stratified on the variable therapy. **Normal typed data**: the entire glioblastoma cohort; **Italic data**: Temozolomide treated glioblastoma patients; **Bold face data**: Glioblastoma patients without temozolomide treatment (Haz. Ratio, Hazard Ratio; Conf. Interval, Confidence Interval).

Since ABCB1 represents a multidrug resistance factor in several malignancies, including glioma [7], we additionally investigated the influence of *ABCB1* promoter methylation on patients’ outcome by using a new established pyrosequencing assay to detect the methylation degree in the *ABCB1* promoter. The analysis of the methylation status involved two CpG sites located in the CpG island of the *ABCB1* promoter and showed a broad interindividual range in the methylation level in our patient cohort with a median of 27.3% (minimum 1.3%, maximum 85.4%). To investigate whether both CpG sites of the *ABCB1* promoter for each person are methylated in the same extent, correlation analysis was performed demonstrating a high correlation of methylation of the two investigated CpG sites (Spearman’s rank correlation coefficient: 0.782, p-value <0.001).

In relation to the OS of all glioblastoma patients and patients treated with temozolomide no significant association of the *ABCB1* methylation status could be detected in a continuous, multivariate Cox model (Table 5). In a cohort of 20 GBM patients, for which ABCB1 expression levels have been available, also no significant correlation between *ABCB1* methylation and expression has been detected (Spearman’s rank correlation coefficient: 0.242, p = 0.304; Figure 1C). However, the *ABCB1* methylation measured in 64 GBM patients was significantly higher than in the controls (Mann Whitney test p = 0.007; Figure 1D), suggesting a different epigenetic regulation in glioblastomas than in healthy brain.

**Table 5:** Multivariate analysis of ABCB1 promoter methylation and its association with the overall survival of GBM patients

**Variable**	**Haz. ratio**	**p-value**	**[95% Conf. Interval]**	
Sex				
male	1.457	0.276	0.740	2.866
(ref. female)	*1.130*	*0.793*	*0.454*	*2.813*
	**4.222**	**0.043**	**1.045**	**17.060**
Age				
50- < 60 years	1.793	0.282	0.619	5.191
(ref. <50 years)	*1.500*	*0.490*	*0.474*	*4.742*
	**5.358**	**0.234**	**0.338**	**84.863**
Age				
60- < 70 years	2.474	0.066	0.942	6.499
(ref. <50 years)	*2.235*	*0.140*	*0.768*	*6.507*
	**3.596**	**0.290**	**0.336**	**38.441**
Age				
≥70 years	6.069	0.001	2.107	17.479
(ref. <50 years)	*9.872*	*0.000*	*2.786*	*34.988*
	**5.112**	**0.167**	**0.505**	**51.721**
Mean methylation level (continuous)				
	0.995	0.461	0.981	1.009
	*1.002*	*0.864*	*0.984*	*1.020*
	**0.973**	**0.032**	**0.950**	**0.998**

Continuous multivariate Cox model regression analysis of ABCB1 promoter methylation and its association with the overall survival (OS) of the analyzed patients with glioblastoma multiforme, adjusted for potential risk factors including sex and age at diagnosis and stratified on the variable therapy. **Normal typed data**: the entire glioblastoma cohort; **Italic data**: Temozolomide treated glioblastoma patients; **Bold face data**: Glioblastoma patients without temozolomide treatment (Haz. Ratio, Hazard Ratio; Conf. Interval, Confidence Interval).

A further resistance factor suggested to be relevant in glioma is the efflux transporter ABCG2 [5]. For determination of the *ABCG2* promoter methylation a novel pyrosequencing assay was established by our group to analyze three CpG sites that have been previously determined in other tumor entities using methylation specific quantitative PCR and bisulfite genomic sequencing [22,23]. The median *ABCG2* promoter methylation status was 30.28% with a broad interindividual range (Min. 3.63%, Max. 83.57%). But for each patient the three investigated *ABCG2* CpG sites show a very high correlation in their methylation degree: CpG site 1 and site 2 with a Spearman’s rank correlation coefficient of 0.972 (p-value <0.0001), CpG site 1 and site 3 with a Spearman’s rank correlation coefficient of 0.953 (p-value <0.0001) and CpG site 2 and site 3 with a Spearman’s rank correlation coefficient of 0.970 (p-value <0.0001).

In continuous multivariate Cox models for all glioblastoma patients (patients treated with and without temozolomide) no trend for a survival benefit has been detected (Table 6). Furthermore, no correlation of *ABCG2* methylation and expression could be identified in a group of 20 GBM patients (Spearman’s rank correlation coefficient: -0.170, p = 0.474; Figure 1E) and no significant difference in *ABCG2* methylation of GBMs and normal brain has been measured (Mann Whitney test p = 0.051; Figure 1F).

**Table 6:** Multivariate analysis of ABCG2 promoter methylation and its association with the overall survival of GBM patients

**Variable**	**Haz. ratio**	**p-value**	**[95% Conf. Interval]**	
Sex				
male	1.463	0.271	0.743	2.879
(ref. female)	*1.092*	*0.842*	*0.459*	*2.600*
	**2.489**	**0.211**	**0.596**	**10.389**
Age				
50- < 60 years	1.745	0.317	0.586	5.195
(ref. <50 years)	*1.454*	*0.545*	*0.433*	*4.882*
	**1.552**	**0.760**	**0.092**	**26.033**
Age				
60- < 70 years	2.270	0.085	0.892	5.778
(ref. <50 years)	*2.271*	*0.118*	*0.811*	*6.358*
	**1.068**	**0.957**	**0.102**	**11.210**
Age				
≥70 years	6.112	0.001	2.087	17.903
(ref. <50 years)	*9.923*	*0.000*	*2.808*	*35.062*
	**2.774**	**0.376**	**0.290**	**26.572**
Mean methylation level (Continuous)				
	1.003	0.736	0.986	1.021
	*0.998*	*0.836*	*0.977*	*1.019*
	**1.018**	**0.430**	**0.974**	**1.065**

Continuous multivariate Cox model regression analysis of ABCG2 promoter methylation and its association with the overall survival (OS) of the analyzed patients with glioblastoma multiforme, adjusted for potential risk factors including sex and age at diagnosis and stratified on the variable therapy. **Normal typed data**: the entire glioblastoma cohort; **Italic data**: Temozolomide treated glioblastoma patients; **Bold face data**: Glioblastoma patients without temozolomide treatment (Haz. Ratio, Hazard Ratio; Conf. Interval, Confidence Interval).

As expected, through all multivariate analyses for both the entire glioblastoma cohort and patients treated with temozolomide a significant worse OS for older patients could be identified.

### Association of the promoter methylation degree with the analyzed Single Nucleotide Polymorphisms (SNPs)

**Figure 2:**
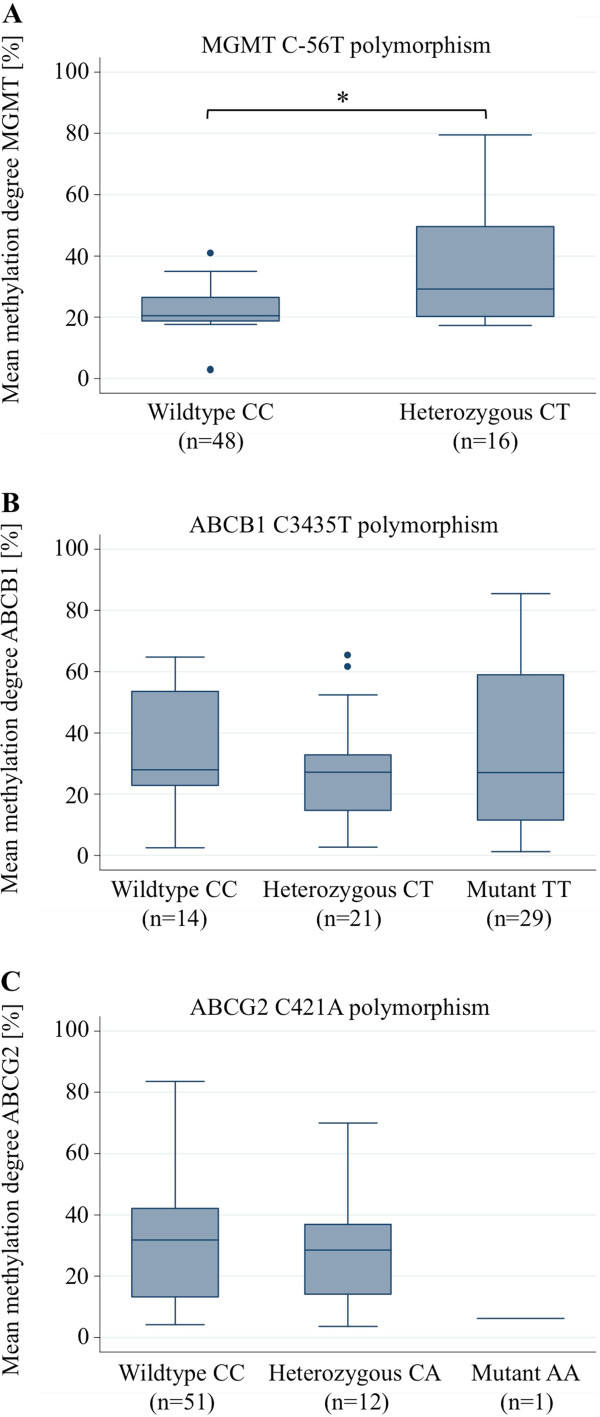
**Relation between MGMT, ABCG2 or ABCB1 promoter methylation and selected SNPs. (A)** Bivariate analysis of the association between the MGMT promoter methylation and the MGMT C-56 T polymorphism (Wilcoxon test, p = 0.02). **(B)** Bivariate analysis of the association between the ABCG2 promoter methylation and the ABCG2 C421A polymorphism (Kruskal-Wallis test, p = 0.30). **(C)** Bivariate analysis of the association between the ABCB1 promoter methylation and the ABCB1 C3435T polymorphism (Kruskal-Wallis test, p = 0.63).

Because of a strong association with the *MGMT* methylation in glioblastoma [24] and further tumors like colorectal carcinoma [25,26], pleural mesothelioma [27], and lung cancer [28], the *MGMT C-56 T* polymorphism was included in our study. The frequency of the *MGMT -56C* and *-56 T* allele was 87.5% and 12.5% in our cohort, respectively, and its distribution was in Hardy-Weinberg equilibrium (p = 0.521). As hypothesized the *MGMT* promoter methylation degree of the analyzed glioblastoma samples was significantly correlated with the genotypes of the *MGMT C-56 T* polymorphism (Figure 2A; Wilcoxon test, p-value = 0.02), showing a higher methylation level in patients with the T allele.

Regarding the analyzed *ABCG2* SNP the frequency of the *ABCG2 421C* and *421A* allele was 89% and 11%, respectively, which is in Hardy-Weinberg equilibrium (p = 0.957) and the frequencies of the *ABCB1* alleles *3435C* and *3435 T* were 38% and 62% in our patient population, respectively, being in Hardy-Weinberg equilibrium with a borderline p-value (p = 0.0503), too. Though the transport function and expression of ABCG2 is known to be influenced by the *ABCG2 C421A* polymorphism [11,12] and the *C3435T* polymorphism in exon 26 seems to modulate the expression of ABCB1 [29], we could not determine an association between the different genotypes of the *ABCG2 C421A* polymorphism and the *ABCG2* promoter methylation (Figure 2C) or for the *ABCB1* methylation status and the *ABCB1 C3435T* polymorphism (Figure 2B).

### Correlation of the methylation degrees between primary tumor and relapse

**Figure 3:**
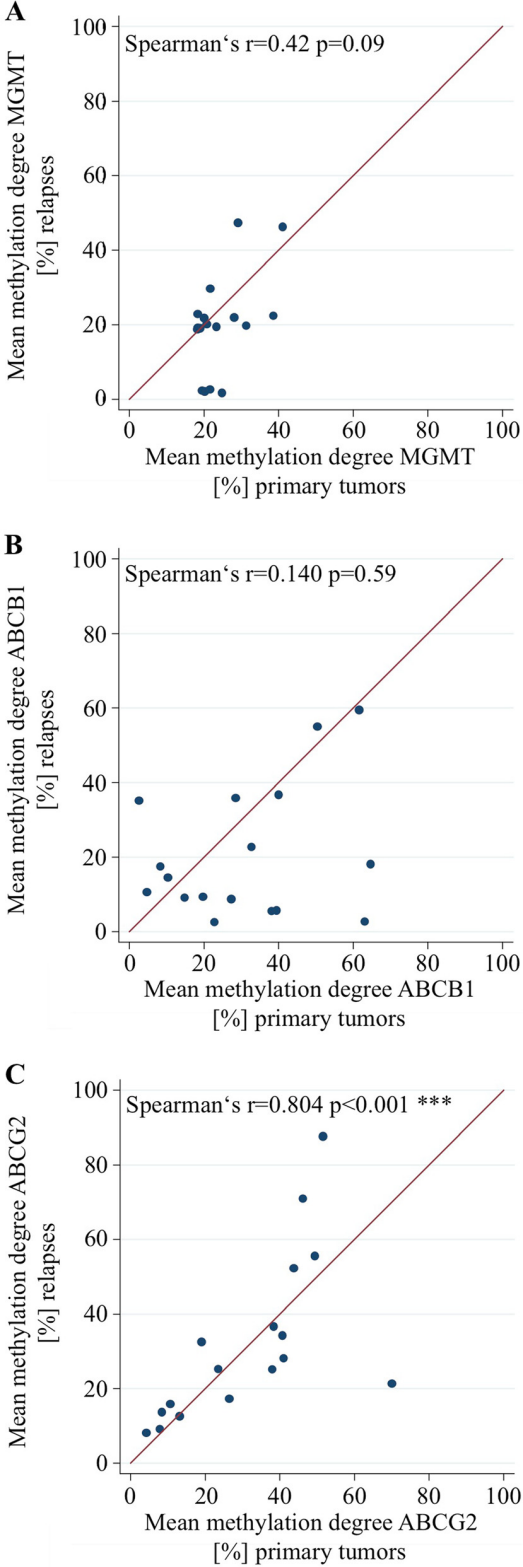
**Correlation analyses of ABCG2, MGMT and ABCB1 promoter methylation in primary tumors and relapses of 17 GBM patients. (A)** Correlation analysis for the median ABCG2 promoter methylation (Spearman’s rank correlation coefficient: 0.804, p < 0.001). **(B)** Correlation analysis for the median MGMT promoter methylation (Spearman’s rank correlation coefficient: 0.42, p = 0.09). **(C)** Correlation analysis for the median ABCB1 promoter methylation (Spearman’s rank correlation coefficient: 0.140, p = 0.59).

To compare the consistency of the methylation degrees before and after treatment, the promoter methylation has been analyzed in 17 primary tumors and relapses of the same patients. The mean *ABCG2* methylation degree of the primary tumors was significantly correlated to the relapses of the respective patients (Spearman’s rank correlation coefficient: 0.804, p-value <0.001; Figure 3C) indicating a stable *ABCG2* promoter methylation level before and after treatment. While the mean *MGMT* methylation degree of the primary tumors showed at least a trend to be correlated to the relapses of the same patients (Spearman’s rank correlation coefficient: 0.42, p-value = 0.09; Figure 3A), for the *ABCB1* methylation status a correlation between primary tumors and relapses was not evident (Figure 3B).

### Relationship of the promoter methylation degree with the age at diagnosis and the gender

Using bivariate analyses, no significant association with the age at diagnosis or the gender has been detected for *MGMT* methylation, *ABCG2* methylation or *ABCB1* methylation (data not shown).

## Discussion

Understanding molecular factors relevant for drug resistance of glioblastoma multiforme is pivotal for the development of personalized therapeutic approaches to this highly aggressive tumor. In several studies the role of *MGMT* methylation as molecular marker for overall survival of glioma patients treated with alkylating agents is discussed [6,17,30,31]. Beside MGMT, the drug efflux transporters ABCB1 and ABCG2 are thought to affect survival of glioma patients due to their role in drug resistance [32,33]. In particular, temozolomide-mediated cytotoxicity is modulated by ABCB1 expression [34]. However, in contrast to *MGMT* methylation no data have existed for *ABCB1* and *ABCG2* promoter methylation in glioblastoma tissue until now. Thus, we focused on establishing new pyrosequencing assays for the analysis of the methylation status of the *ABCB1* and the *ABCG2* promoter in a collective of 64 glioblastoma patients using *MGMT* promoter methylation as reference.

Methylation status was analyzed using pyrosequencing because it allows a highly reproducible quantification of the methylation degree at each individual CpG site and enables rapid parallel processing of a large number of samples [13]. A pivotal role plays the design of the sequencing primer and the pyrosequencing program to minimize the risk of assaying DNA that was not fully converted during bisulfite treatment [13]. However, because pyrosequencing is based on a PCR, which amplifies the bisulfite treated DNA across different epialleles, and the pyrosequencing displays DNA methylation as an average methylation level at each individual CpG position, it is not possible to provide methylation information on an epiallelic level. Thus, results of pyrosequencing should always be interpreted with caution regarding an epiallelic influence.

Compared to pyrosequencing MSP is susceptible to false-positive and false-negative results because of mosaic methylation patterns with variable grade of methylation at the primer positions [13], especially when nested primers are used for clinical samples with small amounts of poor quality DNA like FFPE samples [13,35], which represented the largest proportion of analyzed GBM samples in this study. In addition, Dunn and colleagues described pyrosequencing as suitable method for FFPE samples [19] as well as our fourth tested CpG site of MGMT promoter has been shown as prognostic relevant, while MSP and SQ-MSP for MGMT methylation detection have not been in a Cox model of a recent study [14] and authors recommended pyrosequencing for MGMT methylation analyses in high-throughput settings [36].

In general, in previous studies the role of *MGMT* methylation as molecular marker for overall survival of glioblastoma patients is highly discussed between authors who detected [6,17] or did not find an impact on overall survival [30,31]. We also investigated the previously by Esteller and colleagues published predicting CpG sites [18] but we could not determine a significantly different overall survival of GBM patients (with or without temozolomide treatment) in dependence on their MGMT promoter methylation status. Because this result is contradictory to prior publications about *MGMT* methylation as an independent prognostic factor [6,17,19], we additionally investigated different aspects of the *MGMT* promoter methylation to prove the reliability of our methylation data. Thus, we performed a correlation analysis of *MGMT* promoter methylation and MGMT expression in a subgroup of 20 GBM patients for which MGMT mRNA expression data have been available. A significant negative correlation between *MGMT* promoter methylation and MGMT expression was seen as already predicted by previous studies [20,21]. Furthermore, we found a highly significant elevation of *MGMT* promoter methylation in GBMs compared to normal brain. In agreement with a previous study [19] we also detected only a marginal *MGMT* promoter methylation in non-neoplastic brain samples and a significantly increased *MGMT* promoter methylation in our GBM.

Moreover, we investigated the *MGMT C-56 T* SNP, because it is located in the enhancer region of the *MGMT* gene only 18 bp downstream from the analyzed *MGMT* CpG site. A significantly higher *MGMT* promoter methylation in carriers of the T allele has been described recently in glioblastoma [24], diffuse large B-cell lymphoma [37], colorectal carcinoma [25,26], pleural mesothelioma [27], and lung cancer [28]. In our patient cohort we could confirm a significant higher *MGMT* methylation level in patients with the T allele than in C-56C wildtype patients underlining a precise measurement of the *MGMT* promoter methylation level in our study. Further, this would also imply that patients with the T allele show a minor MGMT expression and thus should have a better response to temozolomide. Contrary to this, we could not find any relation of the *C-56 T MGMT* polymorphism to overall survival of our patient cohort, again arguing against a fundamental role of MGMT in the prognosis of glioblastoma patients as seen by our *MGMT* promoter methylation analysis.

In addition to MGMT, we studied *ABCB1* promoter methylation because ABCB1 is significantly expressed in glioma and discussed as a potential resistance factor [7]. Additionally, for acute lymphocytic leukaemia the methylation of *ABCB1* was associated with a trend toward a better OS [38], while in patients with bronchioloalveolar carcinoma no correlation between *ABCB1* methylation status and patients’ OS was observed [39]. To date, no study analyzing *ABCB1* promoter methylation and its relation to ABCB1 expression and OS of glioblastoma patients is reported. Our new established pyrosequencing assay showed a high correlation of the methylation degree of both analyzed CpG sites with each other similar to the *ABCG2* methylation assay. Despite a significantly higher *ABCB1* methylation in GBM samples of our cohort, the *ABCB1* methylation level was not associated with the OS of GBM patients and was not significantly related to the ABCB1 expression. Similarly, an *ABCB1* promoter hypermethylation was shown in MCF-7 human breast cancer cells [40] and in human prostate cancer compared with benign prostate hypertrophy [41]. Moreover, a significantly higher methylation ratio for the *ABCB1* promoter in gastric cancer samples than for non-neoplastic mucosa has been reported [42]. The prostate cancer study detected a significant correlation of *ABCB1* promoter hypermethylation with worse clinicopathological features [41]. However, both published *ABCB1* methylation studies did not analyze any association with patient’s overall survival.

A further drug resistance gene we decided to analyze was *ABCG2*, because this efflux transporter was found to be expressed in glioma stem cells as well as in endothelial cells of the large vessels of glioma tissue. Similarly to ABCB1, ABCG2 could mediate chemotherapeutic resistance by the efflux of cytostatics [5]. In addition, an inverse correlation between promoter methylation of *ABCG2* and its expression in lung cancer and multiple myeloma has been determined [9,22]. To establish a pyrosequencing assay for *ABCG2* we used a study of Turner and colleagues [22] as reference in order to analyze the same CpG sites in the *ABCG2* promoter, because methylation of these CpG sites was shown to be associated with ABCG2 expression in multiple myeloma. Moreover, a recent study investigated the same CpG sites of the *ABCG2* promoter showing differences in methylation levels between three renal carcinoma cell lines [23].

In our study, a positive correlation of the *ABCG2* methylation level in primary tumor and relapse of the same patient was observed, showing a consistent *ABCG2* methylation status before and after treatment with radio- and chemotherapy. Interestingly, no association between *ABCG2* promoter methylation and ABCG2 expression or overall survival was seen. The missing effect of *ABCG2* methylation on GBM patients’ survival could be explained by the fact that temozolomide, which is the most applied cytostatic for patients with GBM, is not a substrate of ABCG2 [43], and thus modulation of ABCG2 expression should not affect the therapy and survival of GBM patients. Furthermore, for each pyrosequencing assay we assessed a limited number of CpGs (five CpGs for MGMT; two CpGs for ABCB1; three CpGs for ABCG2). Thus, there could be the possibility that CpG sites of the methylation assays, which have not been tested in this study, could have a prognostic value for the GBM patients. However, we interrogated CpG sites, which have been tested in parts before in other publications, as the MGMT CpG sites [6,14,18] and the ABCG2 CpG sites [22] or have been specifically described as prognostic relevant such as our investigated CpG site 4 of the MGMT assay [14]. Furthermore, previous authors investigated a comparable number of CpG sites for MGMT [14]. Nevertheless, it may be useful to test also a larger number of CpG sites for the ABCB1 and ABCG2 assays in the future, e.g. using a HumanMethylation450 (HM-450 K) BeadChip [44].

## Conclusions

In summary, our study represents a combined investigation of promoter methylation and gene polymorphisms of the pivotal drug resistance genes *MGMT*, *ABCB1* and *ABCG2* in glioblastoma multiforme. Our data argue against any relevant impact of *MGMT*, *ABCB1* or *ABCG2* promoter methylation on overall survival of glioblastoma patients. However, we could detect a significant negative correlation between *MGMT* promoter methylation and MGMT expression, a markedly elevated *MGMT* and *ABCB1* promoter methylation in glioblastoma specimens and a significant correlation between *MGMT* methylation and the *MGMT C-56 T* polymorphism.

## Abbreviations

ABC: ATP-binding cassette; MGMT: O^6^-methylguanine-DNA methyltransferase; CpG: Cytidine phosphate guanosine; GBM: Glioblastoma multiforme WHO °IV; PCR: Polymerase chain reaction; WHO: World Health Organization.

## Competing interests

The authors declare that they have no competing interests.

## Authors’ contributions

MCO, SBM, CR, DR, HWSS, and HKK participated in research design. MCO, SBM, KH, SH, HM, JPZ, HB and ANW conducted experiments. MCO, SBM, KW, SV, and WH performed data analysis. MCO, SBM, KW, IC and HKK wrote or contributed to the writing of the manuscript. All authors read and approved the final manuscript.

## Pre-publication history

The pre-publication history for this paper can be accessed here:

http://www.biomedcentral.com/1471-2407/13/617/prepub

## Supplementary Material

Additional file 1 **PCR amplification of promoter regions of interest.Figure S1.** Illustration of the MGMT promoter sequence analyzed by pyrosequencing for determination of the methylation status. **Figure S2.** Illustration of the ABCB1 promoter sequence analyzed by pyrosequencing for determination of the methylation status. **Figure S3.** Illustration of the ABCG2 promoter sequence analyzed by pyrosequencing for determination of the methylation status. PCR-RFLP amplification details. **Figure S4A-F.** Real-Time PCR efficiencies. **Figure S5.** Grading of MGMT methylation levels according to Dunn et al., 2009. **Tables S1A-C.** Quantitative accuracy of methylation assays. **Figure S6.1-4: Figure S6.1.** mRNA expression of CD133, GFAP and PECAM. **Figure S6.2.** Correlation analysis of CD133, GFAP and PECAM with MGMT expression. **Figure S6.3.** Correlation analysis of CD133, GFAP and PECAM with ABCB1 expression. **Figure S6.4.** Correlation analysis of CD133, GFAP and PECAM with ABCG2 expression. **Figure S7.** Comparison of housekeeping genes. **Figure S8** and **Table S2.** Data of Methylation-specific PCR (MSP) for MGMT according to Hegi et al., 2005. Click here for file

## References

[B1] Piperi C Themistocleous MS Papavassiliou GA Farmaki E Levidou G Korkolopoulou P Adamopoulos C Papavassiliou AG High incidence of MGMT and RARbeta promoter methylation in primary glioblastomas: association with histopathological characteristics, inflammatory mediators and clinical outcome Mol Med 2010 13 1–2 1 9 19809523 10.2119/molmed.2009.00140 PMC2757033

[B2] Louis DN Ohgaki H Wiestler OD Cavenee WK Burger PC Jouvet A Scheithauer BW Kleihues P The 2007 WHO classification of tumours of the central nervous system Acta Neuropathol 2007 13 2 97 109 10.1007/s00401-007-0243-4 17618441 PMC1929165

[B3] Reifenberger G Collins VP Pathology and molecular genetics of astrocytic gliomas J Mol Med 2004 13 10 656 670 10.1007/s00109-004-0564-x 15316624

[B4] Rich JN Hans C Jones B Iversen ES McLendon RE Rasheed BK Dobra A Dressman HK Bigner DD Nevins JR Gene expression profiling and genetic markers in glioblastoma survival Cancer Res 2005 13 10 4051 4058 10.1158/0008-5472.CAN-04-3936 15899794

[B5] Bleau AM Huse JT Holland EC The ABCG2 resistance network of glioblastoma Cell Cycle 2009 13 18 2936 2944 19713741

[B6] Hegi ME Diserens AC Gorlia T Hamou MF de Tribolet N Weller M Kros JM Hainfellner JA Mason W Mariani L MGMT gene silencing and benefit from temozolomide in glioblastoma N Engl J Med 2005 13 10 997 1003 10.1056/NEJMoa043331 15758010

[B7] Demeule M Shedid D Beaulieu E del Maestro RF Moghrabi A Ghosn PB Moumdjian R Berthelet F Beliveau R Expression of multidrug-resistance P-glycoprotein (MDR1) in human brain tumors Int J Cancer 2001 13 1 62 66 10.1002/ijc.1306 11391622

[B8] Nakayama M Wada M Harada T Nagayama J Kusaba H Ohshima K Kozuru M Komatsu H Ueda R Kuwano M Hypomethylation status of CpG sites at the promoter region and overexpression of the human MDR1 gene in acute myeloid leukemias Blood 1998 13 11 4296 4307 9834236

[B9] Nakano H Nakamura Y Soda H Kamikatahira M Uchida K Takasu M Kitazaki T Yamaguchi H Nakatomi K Yanagihara K Methylation status of breast cancer resistance protein detected by methylation-specific polymerase chain reaction analysis is correlated inversely with its expression in drug-resistant lung cancer cells Cancer 2008 13 5 1122 1130 10.1002/cncr.23285 18219662

[B10] Jiang ZP Xu P Liu RR Li HD Wang GP Zhao XL Chen FP Correlation between MDR1 methylation status in the promoter region and MDR1 genetic polymorphism in 194 healthy Chinese Han subjects Pharmacogenomics 2008 13 12 1801 1808 10.2217/14622416.9.12.1801 19072639

[B11] Imai Y Nakane M Kage K Tsukahara S Ishikawa E Tsuruo T Miki Y Sugimoto Y C421A polymorphism in the human breast cancer resistance protein gene is associated with low expression of Q141K protein and low-level drug resistance Mol Cancer Ther 2002 13 8 611 616 12479221

[B12] Morisaki K Robey RW Ozvegy-Laczka C Honjo Y Polgar O Steadman K Sarkadi B Bates SE Single nucleotide polymorphisms modify the transporter activity of ABCG2 Cancer Chemother Pharmacol 2005 13 2 161 172 10.1007/s00280-004-0931-x 15838659

[B13] Mikeska T Bock C El-Maarri O Hubner A Ehrentraut D Schramm J Felsberg J Kahl P Buttner R Pietsch T Optimization of quantitative MGMT promoter methylation analysis using pyrosequencing and combined bisulfite restriction analysis J Mol Diagn 2007 13 3 368 381 10.2353/jmoldx.2007.060167 17591937 PMC1899414

[B14] Karayan-Tapon L Quillien V Guilhot J Wager M Fromont G Saikali S Etcheverry A Hamlat A Loussouarn D Campion L Prognostic value of O6-methylguanine-DNA methyltransferase status in glioblastoma patients, assessed by five different methods J Neurooncol 2010 13 3 311 322 10.1007/s11060-009-0031-1 19841865

[B15] Hermes M Geisler H Osswald H Riehle R Kloor D Alterations in S-adenosylhomocysteine metabolism decrease O6-methylguanine DNA methyltransferase gene expression without affecting promoter methylation Biochem Pharmacol 2008 13 11 2100 2111 10.1016/j.bcp.2008.02.031 18395186

[B16] Brescia P Ortensi B Fornasari L Levi D Broggi G Pelicci G CD133 is essential for glioblastoma stem cell maintenance Stem Cells 2013 13 5 857 869 10.1002/stem.1317 23307586

[B17] Weller M Felsberg J Hartmann C Berger H Steinbach JP Schramm J Westphal M Schackert G Simon M Tonn JC Molecular predictors of progression-free and overall survival in patients with newly diagnosed glioblastoma: a prospective translational study of the German Glioma Network J Clin Oncol 2009 13 34 5743 5750 10.1200/JCO.2009.23.0805 19805672

[B18] Esteller M Garcia-Foncillas J Andion E Goodman SN Hidalgo OF Vanaclocha V Baylin SB Herman JG Inactivation of the DNA-repair gene MGMT and the clinical response of gliomas to alkylating agents N Engl J Med 2000 13 19 1350 1354 10.1056/NEJM200011093431901 11070098

[B19] Dunn J Baborie A Alam F Joyce K Moxham M Sibson R Crooks D Husband D Shenoy A Brodbelt A Extent of MGMT promoter methylation correlates with outcome in glioblastomas given temozolomide and radiotherapy Br J Cancer 2009 13 1 124 131 10.1038/sj.bjc.6605127 19536096 PMC2713697

[B20] Qian XC Brent TP Methylation hot spots in the 5′ flanking region denote silencing of the O6-methylguanine-DNA methyltransferase gene Cancer Res 1997 13 17 3672 3677 9288770

[B21] Watts GS Pieper RO Costello JF Peng YM Dalton WS Futscher BW Methylation of discrete regions of the O6-methylguanine DNA methyltransferase (MGMT) CpG island is associated with heterochromatinization of the MGMT transcription start site and silencing of the gene Mol Cell Biol 1997 13 9 5612 5619 9271436 10.1128/mcb.17.9.5612 PMC232409

[B22] Turner JG Gump JL Zhang C Cook JM Marchion D Hazlehurst L Munster P Schell MJ Dalton WS Sullivan DM ABCG2 expression, function, and promoter methylation in human multiple myeloma Blood 2006 13 12 3881 3889 10.1182/blood-2005-10-009084 16917002 PMC1895461

[B23] To KK Zhan Z Bates SE Aberrant promoter methylation of the ABCG2 gene in renal carcinoma Mol Cell Biol 2006 13 22 8572 8585 10.1128/MCB.00650-06 16954373 PMC1636795

[B24] McDonald KL Rapkins RW Olivier J Zhao L Nozue K Lu D Tiwari S Kuroiwa-Trzmielina J Brewer J Wheeler HR The T genotype of the MGMT C>T (rs16906252) enhancer single-nucleotide polymorphism (SNP) is associated with promoter methylation and longer survival in glioblastoma patients Eur J Cancer 2013 13 2 360 368 10.1016/j.ejca.2012.08.012 22975219

[B25] Ogino S Hazra A Tranah GJ Kirkner GJ Kawasaki T Nosho K Ohnishi M Suemoto Y Meyerhardt JA Hunter DJ MGMT germline polymorphism is associated with somatic MGMT promoter methylation and gene silencing in colorectal cancer Carcinogenesis 2007 13 9 1985 1990 10.1093/carcin/bgm160 17621591

[B26] Hawkins NJ Lee JH Wong JJ Kwok CT Ward RL Hitchins MP MGMT methylation is associated primarily with the germline C > T SNP (rs16906252) in colorectal cancer and normal colonic mucosa Mod Pathol 2009 13 12 1588 1599 10.1038/modpathol.2009.130 19734844

[B27] Kristensen LS Nielsen HM Hager H Hansen LL Methylation of MGMT in malignant pleural mesothelioma occurs in a subset of patients and is associated with the T allele of the rs16906252 MGMT promoter SNP Lung Cancer 2011 13 2 130 136 10.1016/j.lungcan.2010.05.008 20627446

[B28] Leng S Bernauer AM Hong C Do KC Yingling CM Flores KG Tessema M Tellez CS Willink RP Burki EA The A/G allele of rs16906252 predicts for MGMT methylation and is selectively silenced in premalignant lesions from smokers and in lung adenocarcinomas Clin Cancer Res 2011 13 7 2014 2023 10.1158/1078-0432.CCR-10-3026 21355081 PMC3070839

[B29] Hoffmeyer S Burk O von Richter O Arnold HP Brockmoller J Johne A Cascorbi I Gerloff T Roots I Eichelbaum M Functional polymorphisms of the human multidrug-resistance gene: multiple sequence variations and correlation of one allele with P-glycoprotein expression and activity in vivo Proc Natl Acad Sci U S A 2000 13 7 3473 3478 10.1073/pnas.97.7.3473 10716719 PMC16264

[B30] Costa BM Caeiro C Guimaraes I Martinho O Jaraquemada T Augusto I Castro L Osorio L Linhares P Honavar M Prognostic value of MGMT promoter methylation in glioblastoma patients treated with temozolomide-based chemoradiation: a Portuguese multicentre study Oncol Rep 2010 13 6 1655 1662 20428822 10.3892/or_00000808

[B31] Martinez R Martin-Subero JI Rohde V Kirsch M Alaminos M Fernandez AF Ropero S Schackert G Esteller M A microarray-based DNA methylation study of glioblastoma multiforme Epigenetics 2009 13 4 255 264 19550145 10.4161/epi.9130

[B32] Lu C Shervington A Chemoresistance in gliomas Mol Cell Biochem 2008 13 1–2 71 80 18259841 10.1007/s11010-008-9722-8

[B33] Andersson U Malmer B Bergenheim AT Brannstrom T Henriksson R Heterogeneity in the expression of markers for drug resistance in brain tumors Clin Neuropathol 2004 13 1 21 27 14986930

[B34] Schaich M Kestel L Pfirrmann M Robel K Illmer T Kramer M Dill C Ehninger G Schackert G Krex D A MDR1 (ABCB1) gene single nucleotide polymorphism predicts outcome of temozolomide treatment in glioblastoma patients Ann Oncol 2009 13 1 175 181 18687982 10.1093/annonc/mdn548

[B35] Derks S Lentjes MH Hellebrekers DM de Bruine AP Herman JG van Engeland M Methylation-specific PCR unraveled Cell Oncol 2004 13 5–6 291 299 15623939 10.1155/2004/370301 PMC4611123

[B36] Christians A Hartmann C Benner A Meyer J von Deimling A Weller M Wick W Weiler M Prognostic value of three different methods of MGMT promoter methylation analysis in a prospective trial on newly diagnosed glioblastoma PLoS One 2012 13 3 e33449 10.1371/journal.pone.0033449 22428052 PMC3302822

[B37] Kristensen LS Treppendahl MB Asmar F Girkov MS Nielsen HM Kjeldsen TE Ralfkiaer E Hansen LL Gronbaek K Investigation of MGMT and DAPK1 methylation patterns in diffuse large B-cell lymphoma using allelic MSP-pyrosequencing Sci Rep 2013 13 2789 24071855 10.1038/srep02789 PMC3784959

[B38] Garcia-Manero G Bueso-Ramos C Daniel J Williamson J Kantarjian HM Issa JP DNA methylation patterns at relapse in adult acute lymphocytic leukemia Clin Cancer Res 2002 13 6 1897 1903 12060634

[B39] Gao P Yang X Xue YW Zhang XF Wang Y Liu WJ Wu XJ Promoter methylation of glutathione S-transferase pi1 and multidrug resistance gene 1 in bronchioloalveolar carcinoma and its correlation with DNA methyltransferase 1 expression Cancer 2009 13 14 3222 3232 10.1002/cncr.24369 19484794

[B40] David GL Yegnasubramanian S Kumar A Marchi VL de Marzo AM Lin X Nelson WG MDR1 promoter hypermethylation in MCF-7 human breast cancer cells: changes in chromatin structure induced by treatment with 5-Aza-cytidine Cancer Biol Ther 2004 13 6 540 548 15034303 10.4161/cbt.3.6.845

[B41] Enokida H Shiina H Igawa M Ogishima T Kawakami T Bassett WW Anast JW Li LC Urakami S Terashima M CpG hypermethylation of MDR1 gene contributes to the pathogenesis and progression of human prostate cancer Cancer Res 2004 13 17 5956 5962 10.1158/0008-5472.CAN-04-0081 15342374

[B42] Tahara T Arisawa T Shibata T Yamashita H Yoshioka D Hirata I Effect of promoter methylation of multidrug resistance 1 (MDR1) gene in gastric carcinogenesis Anticancer Res 2009 13 1 337 341 19331170

[B43] Bleau AM Hambardzumyan D Ozawa T Fomchenko EI Huse JT Brennan CW Holland EC PTEN/PI3K/Akt pathway regulates the side population phenotype and ABCG2 activity in glioma tumor stem-like cells Cell Stem Cell 2009 13 3 226 235 10.1016/j.stem.2009.01.007 19265662 PMC3688060

[B44] Bady P Sciuscio D Diserens AC Bloch J van den Bent MJ Marosi C Dietrich PY Weller M Mariani L Heppner FL MGMT methylation analysis of glioblastoma on the Infinium methylation BeadChip identifies two distinct CpG regions associated with gene silencing and outcome, yielding a prediction model for comparisons across datasets, tumor grades, and CIMP-status Acta Neuropathol 2012 13 4 547 560 10.1007/s00401-012-1016-2 22810491 PMC3444709

