# Supplementary material for: Epigenetic modulation of the drug resistance genes MGMT, ABCB1 and ABCG2 in glioblastoma multiforme
Source: BMC Cancer. 2013 Dec 31;13:617. doi: 10.1186/1471-2407-13-617 (PMC3890604; doi:10.1186/1471-2407-13-617)
Supplement: Additional file 1 — PCR amplification of promoter regions of interest.Figure S1. Illustration of the MGMT promoter sequence analyzed by pyrosequencing for determination of the methylation status. Figure S2. Illustration of the ABCB1 promoter sequence analyzed by pyrosequencing for determination of the methylation status. Figure S3. Illustration of the ABCG2 promoter sequence analyzed by pyrosequencing for determination of the methylation status. PCR-RFLP amplification details. Figure S4A-F. Real-Time PCR efficiencies. Figure S5. Grading of MGMT methylation levels according to Dunn et al., 2009. Tables S1A-C. Quantitative accuracy of methylation assays. Figure S6.1-4: Figure S6.1. mRNA expression of CD133, GFAP and PECAM. Figure S6.2. Correlation analysis of CD133, GFAP and PECAM with MGMT expression. Figure S6.3. Correlation analysis of CD133, GFAP and PECAM with ABCB1 expression. Figure S6.4. Correlation analysis of CD133, GFAP and PECAM with ABCG2 expression. Figure S7. Comparison of housekeeping genes. Figure S8 and Table S2. Data of Methylation-specific PCR (MSP) for MGMT according to Hegi et al., 2005. [file 1471-2407-13-617-S1.docx]

**Additional file S1**

**PCR amplification of promoter regions of interest**

Because *MGMT* methylation forward and reverse primer bind on bisulfite changed sites of the DNA sequence we created the primers with the special wobble mixtures “Y” and “R” instead of one of the four classical bases. The primers have been created to analyze 5 CpG sites of the *MGMT* promoter, which have been investigated before by the cutting-edge publication of Esteller et al. [[1](#_ENREF_1)] (Figure S1). Each 50 µl PCR reaction contained 2 µl of bisulfite treated DNA, 40 µM primers (Invitrogen, Karlsruhe, Germany) and 200 µM dNTPs (Biozym, Hess. Oldendorf, Germany), 1 U Platinum^®^ PCR *Taq* DNA Polymerase, 50 mM TMAC (Tetramethylammoniumchlorid), 10x PCR Buffer without magnesium (200 mM Tris-HCl pH 8.4, 500 mM KCl) and 2 mM MgCl_2_ (all from Invitrogen, Karlsruhe, Germany). The thermal cycling protocol consisted of an initial cycle at 94°C for 3 min followed by 50 cycles at 94°C for 15 sec, 58°C for 30 sec and 72°C for 30 sec, and final extension at 72°C for 7 min.

For *ABCB1* methylation analysis newly designed pyrosequencing primers have been created to detect the methylation of two CpG sites in a CpG island of the *ABCB1* promoter, which contains a high density of CG-rich sequences (Figure S2). Each 50 µl PCR reaction contained 2 µl of bisulfite treated DNA, 64 µM primers (Biomers, Ulm, Germany) and 200 µM dNTPs (Biozym, Hess. Oldendorf, Germany), 1 U Platinum^®^ PCR *Taq* DNA Polymerase, 10x PCR Buffer without magnesium (200 mM Tris-HCl pH 8.4, 500 mM KCl) and 1.5 mM MgCl_2_ (all from Invitrogen, Karlsruhe, Germany). The thermal cycling protocol consisted of an initial cycle at 94°C for 5 min followed by 50 cycles at 94°C for 30 sec, 63°C for 30 sec and 72°C for 30 sec, and final extension at 72°C for 7 min.

For *ABCG2* methylation analysis three CpG sites in the promoter have been chosen for analysis referring to two previous publications [[2](#_ENREF_2), [3](#_ENREF_3)] (Figure S3). Each 50 µl PCR reaction contained 2 µl of bisulfite treated DNA, 50 µM primers (Biomers, Ulm, Germany) and 400 µM dNTPs (Biozym, Hess. Oldendorf, Germany), 1.25 U Platinum^®^ PCR *Taq* DNA polymerase, 10x PCR Buffer without magnesium (200 mM Tris-HCl pH 8.4, 500 mM KCl) and 4 mM MgCl_2_ (all from Invitrogen, Karlsruhe, Germany). The thermal cycling protocol consisted of an initial cycle at 94°C for 3 min followed by 50 cycles at 95°C for 30 sec, 58°C for 30 sec and 72°C for 30 sec, and final extension at 72°C for 10 min.

All PCR amplifications were carried out in a thermal cycler (iCycler iQ5 Real Time PCR Instrument, BioRad, München, Germany). The resultant PCR products were checked by gel electrophoresis to confirm the size of the product.

**Figure S1.** Illustration of the *MGMT* promoter sequence analyzed by pyrosequencing for determination of the methylation status (according to <http://www.ncbi.nlm.nih.gov/nuccore/X61657.1>; Gene: X61657.1).

1008

CTCGGCCCCGCCCCCGCGCCCCGGATATGCTGGGACAGCCCGCGCCCCTAGAA

TTTGGTTTTGTTTTTGTGTTTTGGATATGTTGGGATAGTTTGTGTTTTTAGAA

MGMT forward YGYGTTTYGGATATGTTGGGATAG

MGMT sequencing primer GGATAGTTYGYGTTTTTAGA

Esteller unmethyl. forward primer

Esteller methyl. forward primer

CGCTTTGCGTCCCGACGCCCGCAGGTCCTCGCGGTGCGCACCGTTTGCGACTT

TGTTTTGTGTTTTGATGTTTGTAGGTTTTTGTGGTGTGTATTGTTTGTGATTT

MGMT reverse primer

TTTGTGTTTTGATGTTTGTAGGTTTTTGT Esteller unmethyl.

TTTCGACGTTCGTAGGTTTTCGC Esteller methyl.

1144

GGTGAGTGTCTGGGTCGCCTCGCTCCCGGAA

GGTGAGTGTTTGGGTTGTTTTGTTTTTGGAA

ACTCACAAACCCARCAAARCAA

reverse primer ACAAAACAAAAACCTTCTCACACCTCAA

reverse primer GCAAAGCAAAAGCCTTCTCACG

Legend

Genomic sequence

Bisulfite treated sequence

CpG site

Analyzed CpG site

**Figure S2.** Illustration of the *ABCB1* promoter sequence analyzed by pyrosequencing for determination of the methylation status (according to <http://www.ensembl.org/Homo_sapiens/Gene/Sequence?d> =core;g=ENSG00000085563;r=7:87133175-87342611;t=ENST00000265724; Gene: ENSG00000085563).

113368

AGCCCGCGCGGTGCGGGGACCTGCTCTCTGAGCCCGCGGGCGGTGGGTGGGAG

AGTTTGTGTGGTGTGGGGATTTGTTTTTTGAGTTTGTGGGTGGTGGGTGGGAG

ABCB1 forward primer GTGGGTGGGAG

GAAGCATCGTCCGCGGCGACTGGAACCGGGAGGGAGAATCGCACTGGCGGCGG

GAAGTATTGTTTGTGGTGATTGGAATTGGGAGGGAGAATTGTATTGGTGGTGG

GAAGTAT

ABCB1 sequencing primer GG

GCAAAGTCCAGAACGCGCTGCCAGACCCCCAACTCTGCCTTCGTGGAGATGCT

GTAAAGTTTAGAATGTGTTGTTAGATTTTTAATTTTGTTTTTGTGGAGATGTT

ABCB1 reverse primer CACCTCTACAA

GTAAAGTTTAGAA

113579

GGAGACCCCGCGCACAGGAAAGCCCCTGCAGTGCCCATCGCGGCCAGAGCAGC

GGAGATTTTGTGTATAGGAAAGTTTTTGTAGTGTTTATTGTGGTTAGAGTAGT

CCTCTAAA

Legend

Genomic sequence

Bisulfite treated sequence

CpG site

Analyzed CpG site

Validation reference for bisulfite treatment

**Figure S3.** Illustration of the *ABCG2* promoter sequence analyzed by pyrosequencing for determination of the methylation status (according to

<http://www.ncbi.nlm.nih.gov/nuccore/338858092?fmt_mask=65536>; Gene: AH011213.2)

1873

ATCCACTTTCTCAGAATCCCATTCACCAGAAACCACCCATTTAACTTGCTCTG

ATTTATTTTTTTAGAATTTTATTTATTAGAAATTATTTATTTAATTTGTTTTG

ABCG2 forward primer GAAATTATTTATTTAATTTGTTTTG

ABCG2

Turner methylated

Turner unmethylated

GGTGCGAGCAGCGCTTGTGACTGGGCAACCTGTGCGTCAGCGTCCCCGGTGCT

GGTGTGAGTAGTGTTTGTGATTGGGTAATTTGTGTGTTAGTGTTTTTGGTGTT

GGTG

seq. primer TTGTGATTGGGTAATTTGTG

forward primer TGATTGGGTAATTTGTGCGTTAGCG

forward primer TGATTGGGTAATTTGTGTGTTAGTGTT

TCGGCGCTCCGGCCAGTGACGGCGACCAAACCCAGCTAGGTCAGACGAGGTAC

TTGGTGTTTTGGTTAGTGATGGTGATTAAATTTAGTTAGGTTAGATGAGGTAT

ABCG2 reverse primer CTCCATA

Turner methylated

TGATCAGCCCAATGAGCGCCTGGTGATTCTCGTAGTTAATCACTCTGGTTCAT

TGATTAGTTTAATGAGTGTTTGGTGATTTTTGTAGTTAATTATTTTGGTTTAT

ACTAATCAAATTACTC

and unmethylated rev. primer CATCAATTAATAAAACCAAATA

2086

TC

TT

AA

Legend

Genomic sequence

Bisulfite treated sequence

CpG site

Analyzed CpG site

**PCR-RFLP amplification details**

For *MGMT* variant analysis, each 25 µl PCR reaction contained about 200 ng DNA, 100 µM primers (Eurofins MWG Operon, Ebersberg, Germany) and 200 µM dNTPs (Biozym, Hess. Oldendorf, Germany), 0.5 U Platinum^®^ PCR *Taq* DNA Polymerase (Invitrogen, Karlsruhe, Germany), 10x PCR Buffer without magnesium (200 mM Tris-HCl pH 8.4, 500 mM KCl) (Invitrogen, Karlsruhe, Germany) and 1.5 mM MgCl_2_. The thermal cycling protocol consisted of an initial cycle at 94°C for 5 min followed by 45 cycles at 95°C for 30 sec, 63°C for 30 sec and 72°C for 30 sec, and final extension at 72°C for 5 min. *MGMT C-56T* PCR products were digested with *Rsa*I endonuclease (10 U) at 37°C overnight (Fermentas, St. Leon-Rot, Germany) and restriction fragments were identified by 2% agarose gel electrophoresis.

For *ABCB1* variant analysis, each 25 µl PCR reaction contained about 100 ng DNA, 40 µM primers and 200 µM dNTPs (Biozym, Hess. Oldendorf, Germany), 0.75 U *Taq* DNA Polymerase (Invitrogen, Karlsruhe, Germany), 10x PCR Buffer without magnesium (200 mM Tris-HCl pH 8.4, 500 mM KCl) (Invitrogen, Karlsruhe, Germany) and 2 mM MgCl_2_. The thermal cycling protocol consisted of an initial cycle at 94°C for 2 min followed by 35 cycles at 94°C for 30 sec, 60°C for 1 min and 72°C for 1 min, and final extension at 72°C for 7 min. *ABCB1 C3435T* PCR products were digested with *Mbo*I endonuclease (5 U) at 37°C overnight (Fermentas, St. Leon-Rot, Germany) and restriction fragments were identified by 2% agarose gel electrophoresis.

For *ABCG2* variant analysis, each 25 µl PCR reaction contained 100 ng DNA, 40 µM primers (Invitrogen, Karlsruhe, Germany) and 400 µM dNTPs (Biozym, Hess. Oldendorf, Germany), 0.5 U Platinum^®^ PCR *Taq* DNA Polymerase (Invitrogen, Karlsruhe, Germany), 10x PCR Buffer without magnesium (200 mM Tris-HCl pH 8.4, 500 mM KCl) (Invitrogen, Karlsruhe, Germany) and 4 mM MgCl_2_. The thermal cycling protocol consisted of an initial cycle at 94°C for 3 min followed by 35 cycles at 94°C for 30 sec, 53°C for 30 sec and 72°C for 30 sec, and final extension at 72°C for 7 min. *ABCG2 C421A* PCR products were digested with *HpyCH4*III endonuclease (2.5 U) at 37°C overnight (New England Biolabs Inc., MA, USA) and restriction fragments were identified by 2% agarose gel electrophoresis.

**Figure S4A-F.** Real-Time PCR efficiencies

**Figure S5.** Grading of MGMT methylation levels according to Dunn et al., 2009

**Tables S1A-C.** Quantitative accuracy of methylation assays

| **Table S1A** | ABCB1 – CpG site 1.1 | ABCB1 – CpG site 1.2 | ABCB1 – CpG site 1.3 |
| --- | --- | --- | --- |
| ABCB1 – CpG site 1.1 |  | 0.944 *** | 0.988 *** |
| ABCB1 – CpG site 1.2 | 0.944 *** |  | 0.956 *** |
| ABCB1 – CpG site 1.3 | 0.988 *** | 0.956 *** |  |

| **Table S1B** | ABCG2 – CpG site 1.1 | ABCG2 – CpG site 1.2 | ABCG2 – CpG site 1.3 |
| --- | --- | --- | --- |
| ABCG2 – CpG site 1.1 |  | 0.963 *** | 0.967 *** |
| ABCG2 – CpG site 1.2 | 0.963 *** |  | 0.979 *** |
| ABCG2 – CpG site 1.3 | 0.967 *** | 0.979 *** |  |

| **Table S1C** | MGMT – CpG site 1.1 | MGMT – CpG site 1.2 | MGMT – CpG site 1.3 |
| --- | --- | --- | --- |
| MGMT – CpG site 1.1 |  | 0.927 *** | 0.878 *** |
| MGMT – CpG site 1.2 | 0.927 *** |  | 0.943 *** |
| MGMT – CpG site 1.3 | 0.878 *** | 0.943 *** |  |

**Figure S6.1-4**

**Figure S6.1.** mRNA expression of CD133, GFAP and PECAM

**Figure S6.2.** Correlation analysis of CD133, GFAP and PECAM with MGMT expression

**

**Figure S6.3.** Correlation analysis of CD133, GFAP and PECAM with ABCB1 expression

**Figure S6.4.** Correlation analysis of CD133, GFAP and PECAM with ABCG2 expression

**Figure S7.** Comparison of housekeeping genes

**Figure S8.** Results of the Methylation-specific PCR (MSP) for MGMT of control DNA (**A**, methylated vs. unmethylated) and selected GBM samples (**B**) according to PCR conditions of Hegi et al. (2005)

**Table S2.** MGMT promoter methylation data of selected samples analyzed by Pyrosequencing

| Sample | Methylation [%] according to Pyrosequencing |
| --- | --- |
| LN18 | 5.61 |
| U87MG | 79.8 |
| GBM1 | 28.2 |
| GBM2 | 13.2 |
| GBM3 | 61.2 |
| GBM4 | 5.57 |
| GBM5 | 3.01 |
| GBM6 | 74.7 |

**References**

1. Esteller M, Garcia-Foncillas J, Andion E, Goodman SN, Hidalgo OF, Vanaclocha V, Baylin SB, Herman JG: **Inactivation of the DNA-repair gene MGMT and the clinical response of gliomas to alkylating agents**. *N Engl J Med* 2000, **343**(19):1350-1354.

2. To KK, Zhan Z, Bates SE: **Aberrant promoter methylation of the ABCG2 gene in renal carcinoma**. *Mol Cell Biol* 2006, **26**(22):8572-8585.

3. Turner JG, Gump JL, Zhang C, Cook JM, Marchion D, Hazlehurst L, Munster P, Schell MJ, Dalton WS, Sullivan DM: **ABCG2 expression, function, and promoter methylation in human multiple myeloma**. *Blood* 2006, **108**(12):3881-3889.
